# Comparison of MRI Features of Epithelioid Hepatic Angiomyolipoma and Hepatocellular Carcinoma: Imaging Data From Two Centers

**DOI:** 10.3389/fonc.2018.00600

**Published:** 2018-12-12

**Authors:** Weihai Liu, Jiawei Wang, Qiang Huang, Qinyan Lu, Wenjie Liang

**Affiliations:** ^1^Department of Radiology, The People's Hospital of Beilun District, Ningbo, China; ^2^Department of Radiology, The Second Affiliated Hospital, Zhejiang University School of Medicine, Hangzhou, China; ^3^Department of Radiology, The First Affiliated Hospital, Zhejiang University School of Medicine, Hangzhou, China; ^4^Department of Radiology, Hangzhou Aeromedicine Evaluation and Training Center of the PLA Air Force, Hangzhou, China

**Keywords:** hepatic angiomyolipoma, CT, MRI, imaging, diagnosis

## Abstract

**Introduction:** Epithelioid hepatic angiomyolipoma (Epi-HAML) can easily be misdiagnosed as a malignant tumor such as hepatocellular carcinoma (HCC) because of the low-fat content on imaging. We analyzed and compared the magnetic resonance imaging (MRI) features of Epi-HAML and HCC, which would aid in disease diagnosis.

**Methods:** We included 30 pathologically confirmed patients with Epi-HAML and 80 with HCC, who underwent both MRI unenhanced scan and three-phase contrast-enhanced MRI scan. The clinical and MRI features of the two groups were summarized and analyzed.

**Results:** Epi-HAML showed significant differences compared to HCC group in terms of clinical features such as sex preference, age, concomitant diseases (hepatitis B and cirrhosis), and elevated plasma alpha-fetoprotein (AFP) (*P* < 0.001). In addition, there were statistically significant differences between both tumor types with regard to conventional MRI findings such as a solitary tumor (100 vs. 83.8%, *P* = 0.018), well-defined (93.3 vs. 71.3%, *P* = 0.027), mild hyperintensity (40.0 vs. 3.7%, *P* < 0.001) on DWI with high b-value, fat within the tumor (43.3 vs. 8.8%, P < 0.001), and rare necrosis (3.3 vs. 26.3%, *P* = 0.016). Besides, Epi-HAML displayed significant differences compared to HCC in terms of contrast-enhanced MRI characteristics such as draining hepatic vein (30.0 vs. 3.8%, *P* < 0.001), portal vein tumor thrombus (0 vs. 13.8%, *P* = 0.033), hypointensity at delayed phase (70.0 vs. 95%, *P* = 0.001), intra-tumor vessel at delayed phase (36.7 vs. 10.0%, *P* = 0.003), pseudocapsule (20.0 vs. 78.8%, *P* < 0.001), and prolonged enhancement (56.7 vs. 1.2%, *P* < 0.001).

**Conclusion:** Epi-HAML frequently occurs in middle-aged women and usually lacks characteristic clinical symptoms. Typically, Epi-HAML presents as an isolated and well-defined tumor with rich vasculature. Specific MRI features such as intra-tumor fat, intra-tumor vessel, draining hepatic vein, prolonged enhancement, and lack of capsule may contribute to a more confident diagnosis of Epi-HAML.

## Introduction

Epithelioid angiomyolipoma (Epi-AML) is a rare type of angiomyolipoma. Distinct from the classical AML, Epi-AML is a tumor with malignant potential; histopathologically, its most dominant feature is the presence of epithelioid smooth muscle cells and perivascular epithelioid cells (1). Therefore, AML was recognized as a member of the neoplasms with perivascular epithelioid cell (PEComas) family in 2002 (2). This classification also includes mesenchymal tumors with similar morphological and immunohistological features in different sites such as the respiratory, digestive, and urogenital systems (3). Epi-AML was first reported by Martignoni et al. (1) in 1995. Thereafter, scattered cases have been reported mainly concentrating on the kidney, followed by the liver.

The pathogenesis of Epi-HAML is unclear so far. Typically, 6.2% Epi-HAML cases are related to the development of gene mutations in tuberous sclerosis syndrome (TSC) (4), while 26.7% cases with renal Epi-AML are related to TSC (5). Epi-HAML frequently occurs in women, with the female-to-male incidence being 5:1 (4, 6). About a half of all Epi-HAML cases are asymptomatic, and others show non-specific symptoms such as abdominal pain, abdominal discomfort, and abdominal distension (6). Previous studies indicate that Epi-HAML can very rarely be diagnosed preoperatively, and about 60% cases are misdiagnosed as hepatocellular carcinoma (HCC). In addition, some Epi-HAML cases are homogeneously misdiagnosed as hepatic adenoma, focal nodular hyperplasia (FNH), and metastatic tumors (6). To definitely diagnose Epi-HAML, it is necessary to perform histopathological and immunohistochemical examinations of the surgically resected or biopsied tumor specimens (4, 7). Classical AML cases are usually treated by non-surgical methods (8), while surgical resection may be an appropriate treatment for Epi-HAML owing to its potential malignant nature (6, 9). Thus, clinicians should clearly distinguish Epi-HAML from malignant tumors such as HCC to avoid unsuitable therapeutic schemes such as transhepatic arterial chemotherapy and embolization (TACE) and liver transplantation.

Imaging examination, particularly contrast-enhanced computed tomography (CT) and magnetic resonance imaging (MRI), are important tools for the clinical evaluation of liver tumors. There is a series of studies reporting the imaging findings of HAML (10–18). Specifically, the features of a well-defined mass with rich blood supply, intra-tumor fat, and tortuous blood vessels are indicative of the imaging diagnosis of HAML (15–18). Distinct from classical HAML, Epi-HAML has low fat content, which is therefore easily confused with malignant tumors like HCC (6). So far, only a few studies have reported the imaging findings of Epi-HAML (6, 7, 19–23). However, these studies are generally individual case reports or reports with a small sample size. To our best knowledge, related imaging data are insufficient, especially regarding the MRI features of Epi-HAML.

Therefore, this study aimed to retrospectively analyze the MRI findings of Epi-HAML. In our study, the clinical and MRI features of patients with Epi-HAML were compared with those of patients with HCC (the control group) to enhance our understanding of Epi-HAML and improve diagnostic accuracy. To our best knowledge, our study has reported the MRI findings of the largest number of Epi-HAML patients thus far.

## Materials and Methods

### Patients

This retrospective study was approved by the Ethics Committee of the First Affiliated Hospital, Zhejiang University School of Medicine; informed consent was obtained for all patients. The pathological diagnosis databases in two institutions (the First Affiliated Hospital, Zhejiang University School of Medicine [Institution I] and the Second Affiliated Hospital, Zhejiang University School of Medicine [Institution II]) were retrieved from January 2010 to December 2017 using the search terms—*hepatic angiomyolipoma* and *epithelioid hepatic angiomyolipoma*. Cases simultaneously conforming to all the following criteria were suitable for enrolment: (i) patients with pathologically confirmed diagnosis of primary Epi-HAML based on biopsied or surgically resected specimens; (ii) Epi-HAML cases with preoperative or preprocedurally unenhanced MRI scan and three-phase contrast-enhanced MRI scan; and (iii) Epi-HAML cases with complete clinicopathological and MRI data for summarization and re-evaluation. The exclusion criteria for Epi-HAML cases were as follows: (i) cases diagnosed with Epi-HAML clinically or radiologically, but lacking relevant pathological results; and (ii) patients with missing MRI data that could not be re-evaluated. The patient inclusion criteria for the HCC group in this study were as follows: (i) patients undergoing surgical resection of the tumor with a pathological diagnosis of primary HCC; (ii) patients receiving preoperative MRI plain scan and contrast-enhanced examination; and (iii) HCC cases with complete clinical pathology and MRI data for re-evaluation. The exclusion criteria for the HCC group were as follows: (i) cases with clinical diagnosis of HCC but with no surgical pathological evidence; (ii) patients receiving treatment such as TACE in preoperative MRI examination; and (iii) HCC cases with incomplete clinical or imaging data.

A total of 30 Epi-HAML cases from Institution I (*n* = 26) and Institution II (*n* = 4) were enrolled in our study. Additionally, 13 Epi-HAML cases were excluded owing to the lack of MRI examination and 2 cases, owing to missing images. A total of 80 HCC cases (Institution I, *n* = 80) were randomly selected during the research period as controls. The clinical data of both groups were collated by one radiologist.

### MRI Scan Protocol

Patients underwent MRI examinations at both institutions on the clinical MRI scanner (3.0 T Signa HDx, GE Medical Systems, Milwaukee, WI, USA, Institution I; 3.0 T Discovery MR750, GE Healthcare, Chicago, IL, USA, Institution II) by using the trunk phased array coil. The scanning sequence parameters before and after contrast-enhanced MRI in the two medical institutions are shown in Table 1. The diffusion weighted image (DWI) sequence was obtained, with the b-values of 1,000 s/mm^2^ in Institution I and 800 s/mm^2^ in Institution II used as the conventional setting. In contrast-enhanced MRI, the contrast agent gadolinium-diethylenetriamine penta-acetic acid (Gd-DTPA, Magnevist, Bayer HealthCare, Berlin, Germany) at a dose of 0.1 mmol/kg was intravenously injected using the high-pressure injection pump. All patients underwent dynamic contrast-enhanced T1 weighted MRI scan after contrast-agent administration, as well as after 14–20 s (hepatic arterial phase), 40–55 s (portal phase), and 120 s (delayed phase).

**Table 1:** Liver MRI scanning parameters in two medical institutions.

	**Institution I**	**Institution II**	**Institution I**	**Institution II**	**Institution I**	**Institution II**	**Institution I**	**Institution II**
Imaging	Non-contrast and contrast T1-weighted image		T2-weighted image		Diffusion-weighted image		Lipid-suppression image	
Sequence	Liver acceleration volume acquisition		Fast spin-echo		Spin-echo/echo planar imaging		Liver acceleration volume acquisition-Flex	
Breath	Breath-hold		Respiratory gating		Respiratory gating		Breath-hold	
Position	Axial	Axial	Axial and coronal	Axial	Axial	Axial	Axial	Axial
TR (ms)	3.3	3.9	8,000	10,000	9000	6,000	4.1^*^/4.1^†^	4.0^*^/4.0^†^
TE (ms)	1.5	1.8	85	88.5	80	52.4	1.1^*^/2.3^†^	1.2^*^/2.4^†^
Flip angle (°)	10	12		-		-	10	12
Matrix size (pixels)	320 × 256	320 × 320	320 × 224	320 × 320	140 × 128	128 × 128	300 × 224	320 × 320
Field of view (cm)	38 × 30.4	38 × 38	38 × 28.5	38 × 38	38 × 28.5	38 × 38	38 × 30.4	38 × 38
NEX	1	1	2	2	2	1	1	1

*Institution I: The First Affiliated Hospital, Zhejiang University School of Medicine; Institution II: The second Affiliated Hospital, Zhejiang University School of Medicine*;

* *parameters of out phase*;

† *parameters of in phase*.

### MRI Image Analysis

Two experienced abdominal radiologists (QH and JWW with 12 and 13 years of imaging diagnosis experience, respectively) blinded to the study design were invited to independently assess the MRI findings of all enrolled cases. The evaluation included tumor site (left hepatic lobe, right hepatic lobe, or caudate lobe; Supplementary Figure 1); size (maximum cross-sectional diameter, <3 or ≥3 cm); morphology (regular or irregular); border (well-defined or ill-defined); and tumor signal intensity. During pre-enhanced imaging, the signal intensities of the tumor on T1WI and T2WI sequences were recorded as hypointense, isointense, or (slight) hyperintense compared with the signal intensity of the surrounding liver parenchyma. Meanwhile, the signal consistency of the tumor was also evaluated on T2WI sequence (homogeneous or heterogeneous). On DWI sequence with *b* = 1,000 (or 800) s/mm^2^, the signal intensity of the lesion was recorded as hypointense, isointense, mild, moderate, or markedly hyperintense based on the experience of the two radiologists. Besides, the evaluated items also included whether there was fat, bleeding, and/or necrotic changes in the tumor.

On contrast-enhanced MRI, the enhanced degree of all lesions was classified as hypoenhancement, isoenhancement, and hyperenhancement (lesion intensity was compared with the surrounding liver parenchyma). The Epi-HAML and HCC MRI images were evaluated during each specific phase and judged based on the signal intensity of majority of the tumor. In addition, the signs of draining hepatic vein, intra-tumor vessel, and abnormal perfusion during arterial phase were also evaluated. During portal phase and delayed phase, the intra-tumor vessel, capsule, and portal venous tumor thrombosis were also assessed. Subsequently, the tumor enhancement pattern was evaluated, which was categorized as wash in and wash out, prolonged enhancement, fading, and poor blood supply. Furthermore, the MRI features of Epi-HAML were defined with reference to the definitions and annotations in the Liver Imaging Reporting and Data System (LI-RADS)^1^.

Among them, intra-tumoral fat was defined as reduced outphase sequence tumor signal compared with the inphase sequence signal. Draining hepatic vein was defined as the specific hepatic vein of the hepatic segment with Epi-HAML displayed during arterial phase, which could be one branch or two. Intra-tumor vessel was defined as the presence of dotted or tortuous branched hyperenhancement in the tumor, which could be confirmed through the continuous imaging sequence. Prolonged enhancement was defined as severe enhancement of the tumor during arterial phase, along with locoregional or overall hyperintensity of the tumor compared with the surrounding liver parenchyma during the portal and delayed phases. Poor blood supply was defined as the tumor manifesting as a lowly enhanced lesion at the three enhanced phases compared with the surrounding liver parenchyma tissues. Any disagreement in imaging evaluation results was discussed to reach a final consensus. Finally, consistent results of the MRI features of the Epi-HAML and HCC groups were obtained.

### Pathological Diagnosis of Epi-HAML and HCC

Paraffin-embedded tumor tissue sections were stained with hematoxylin-eosin (H&E) for histopathological examination. The diagnostic criteria of Epi-HAML were as follows: the tumor comprised three components including blood vessels, smooth muscle-like cells, and adipocytes, with perivascular epithelioid cells (PECs) being dominant; fat component being generally <10%; PECs were round or polygon-shaped, the central cytoplasm was eosinophilic red, while the peripheral cytoplasm was bright, also called as spider cells; and the nuclei were frequently distributed around the eosinophilic red cytoplasm, which were round, oval, or irregular in shape. Tumor cells were usually distributed in a strip or nested manner, with sinusoid cavity lining the endothelium in the center.

The immunohistochemical staining method was extensively illustrated in previous studies (24). In brief, each sample was sliced into 4-μm-thick sections, followed by deparaffinization in xylene and hydration in a graded series of ethanol. Subsequently, the sections were subjected to compression and heating in sodium citrate hydrochloric acid buffer (10 mmol/L, pH 6.0) for antigen repair. Endogenous peroxidase activity was blocked using 0.3% H_2_O_2_ for 10 min, followed by culturing in 5% serum. Afterwards, the sections were incubated with primary antibodies at 37°C for 1.5 h, washed with phosphate buffer solution (PBS), and filtered. Then, horse radish peroxidase (HRP)-conjugated rabbit anti-mouse antibody was placed onto the glass slides, and 3,3-diaminobenzidine (DAB) developing agent was used for developing. All glass slides were counter-stained with hematoxylin. The primary antibodies in non-immune serum were replaced in the experiment to produce the negative control.

Immunohistochemistry indicated that Epi-HAML tissues mainly expressed the chromatophore markers including HMB45 and Melan-A, but not S-100. In addition, some cases could express the myogenic markers such as desmin, SMA, and calponin. Differential diagnosis indicated that Epi-HAML within the liver should be mainly distinguished from HCC (HCC could express hepatocyte and GPC-3 but not HMB45 and Melan-A). Additionally, Epi-HAML should be distinguished from leiomyoma, as leiomyoma could express the myogenic markers but not chromatophore markers like HMB45.

The diagnostic criteria of HCC are as listed: atypical tumor cells that are trabecular, with increased nucleus-to-cytoplasm ratio and pseudoglandular or solid nested arrangement and showing infiltrative growth. Immunohistochemistry suggested that the tumor cells were Hep+, with most tumor cells being GPE3+; CD34 displayed capillarization of sinusoidal endothelium, and net staining suggested hepatic plate widening and destruction. All tissue specimens were evaluated by two experienced pathologists with 12 and 11 years of diagnosis experience, respectively.

### Data Statistics

The mean and standard deviation (SD) of age were calculated, and the distribution of age as well as tumor diameter was analyzed. The clinical features, conventional MRI findings, and contrast-enhanced MRI characteristics were analyzed by chi-square test or Fisher's exact test using the SPSS software (Version 23.0; SPSS Inc., Chicago, IL, USA). The chi-square test was used for analysis the sex; symptoms; hepatitis B status; smoking and alcohol abuse; size, shape, border, tumor intensity on T2-weighted imaging, and fat content in conventional MRI features; intra-tumor vessel at arterial and delayed phases; abnormal perfusion; tumor intensity at portal phase; and pseudocapsule in contrast-enhanced MRI characteristics. Chi-square test was not appropriate for cell (with the theoretical frequency of <1 or <5) proportion of over 20%, and thus Fisher's exact test was used to compare other features. A difference of *P* < 0.05 was deemed statistically significant, while a difference of P < 0.01 was considered markedly statistical significance.

## Results

### Clinical Features of Epi-HAML vs. HCC

Among the 30 Epi-HAML cases, 22 were women and eight were men. The age of onset ranged from 27 to 67 years (mean age, 47.87 ± 10.33 years). Twenty-one of the above 30 Epi-HAML cases showed no clinical symptoms (including 18 cases being discovered through routine check-up), while the remaining nine showed clinical symptoms such as abdominal pain (*n* = 5), abdominal distention (*n* = 3), and abdominal discomfort (*n* = 1). With regard to concomitant diseases, four cases had hepatitis B virus (HBV) infection, four had fatty liver disease, one had cholelithiasis, and three had hypertension. As for lifestyle habits, five patients consumed alcohol and four were smokers. In this study, no Epi-HAML case was accompanied with liver cirrhosis, TSC, or elevated plasma AFP.

There were 80 HCC patients in the control group, including 10 women and 70 men, with the age of onset being 23–83 years (mean age, 61.00 ± 10.07 years). Twenty-seven of these 80 HCC patients showed no clinical symptoms and were discovered through general health examination or clinical screening; while the remaining 53 cases displayed various abdominal or systemic symptoms, such as hepatalgia (*n* = 21), abdominal pain (*n* = 15), abdominal distention (*n* = 7), and other symptoms (*n* = 10, such as abdominal mass, gastrointestinal bleeding, weakness, and fever). As for concomitant diseases, 72 cases had hepatitis B virus (HBV) infection, 54 had liver cirrhosis, five had fatty liver, nine had cholelithiasis, eight had hypertension, and seven had diabetes. With regard to lifestyle choices, 35 patients were smokers and 25 were alcoholic. In this study, 55 HCC cases had abnormally elevated plasma AFP. All clinical features of the two groups are summarized in Table 2.

**Table 2:** Comparison of the clinical features of epithelioid hepatic angiomyolipoma versus hepatocellular carcinoma.

	**Epi-HAML (*n* = 30)**	**HCC (*n* = 80)**	***p***
Sex			<0.001
Male	8 (26.7%)	70 (87.5%)	
Female	22 (73.3%)	10 (12.5%)	
Age (years)	47.87 ± 10.33	61.00 ± 10.07	<0.001
Symptoms			0.001
Asymptomatic	21 (70.0%)	27 (33.8%)	
Symptomatic	9 (30.0%)	53 (66.2%)	
Concomitant disease			
Hepatitis B	4 (13.3%)	72 (90.0%)	<0.001
Cirrhosis	0	54 (67.5%)	<0.001
Fatty liver disease	4 (13.3%)	5 (6.3%)	0.414
Cholelithiasis	1 (3.3%)	9 (11.3%)	0.361
Hypertension	3 (10.0%)	8 (10.0%)	1
Diabetes	0	7 (8.8%)	0.006
Personal habits			
Smoking	4 (13.3%)	35 (43.8%)	0.006
Alcohol abuse	5 (16.7%)	25 (31.3%)	0.197
Elevated plasma AFP	0	55 (68.8%)	<0.001

*Epi-HAML, epithelioid hepatic angiomyolipoma; HCC, hepatocellular carcinoma; AFP, alpha-fetoprotein*.

There were markedly significant differences in the clinical features such as sex, age, symptoms, concomitant diseases (hepatitis B and liver cirrhosis), and abnormally elevated plasma AFP between the Epi-HAML and HCC groups (*P* < 0.001). Second, concomitant disease (diabetes) and personal habits (smoking) between the Epi-HAML and HCC groups were markedly statistically significant (*P* < 0.01). No other clinical features were significantly different between the two groups. The comparisons of all clinical features between both groups are summarized in Table 2.

### Conventional MRI Features of Epi-HAML vs. HCC

In this study, all 30 Epi-HAML cases had solitary tumors, with the axial maximum diameter ranging from 0.9 to 7.4 cm (including 20 cases of ≥3 cm) and the average of 3.86 ± 1.90 cm. In terms of tumor site, 18 cases were located in the right lobe, 10 cases in the left lobe, and two cases in the caudate lobe. As for tumor shape, 22 lesions were regular in morphology, which were quasi-circular or oval, while the remaining eight were irregular. Meanwhile, the border in 28 cases were well-defined, whereas two cases showed ill-defined borders.

In the Epi-HAML group, 29 cases showed hypointensity, while one case showed isointensity on T1WI. On T2WI, 28 cases displayed slight hyperintensity, and two cases showed isointensity. Among them, 13 cases showed homogeneous signal, while 17 cases displayed heterogeneous signal. Besides, only 25 cases underwent DWI sequence scan, including two cases with hypointensity, 10 cases with mild hyperintensity, eight cases with moderate hyperintensity, and five cases with marked hyperintensity at the high b value. Moreover, the fat component signal within the tumor was confirmed in 13 cases, bleeding in two cases and necrosis in one case.

Among the 80 HCC cases in the control group, 67 had solitary HCC, while 13 had multiple HCC, with the axial maximum tumor diameter of 0.8–12.0 cm (mean diameter, 5.27 ± 3.36 cm; 57 cases had tumor diameter ≥ 3 cm). Forty-nine cases had tumors at the right lobes, 30 at the left lobes, and one at the caudate lobe. Morphologically, 59 tumors showed regular morphology, in that they were circular, quasi-circular, or oval; while the remaining 21 lesions showed irregular morphology. Moreover, 57 tumors were well-defined and 23 were ill-defined. On T1WI, 78 cases showed hypointensity, and 2 revealed isointensity. On T2WI, 79 cases manifested as slight hyperintensity, while 1 as isointensity. In addition, 25 tumors showed homogeneous signals on T2WI, while 55 showed heterogeneous signals. All the 80 tumors manifested as hyperintense lesions on the DWI sequence with high b-value, of which, three had mild hyperintensity, 15 had moderate hyperintensity, and 62 had marked hyperintensity. When confirmed by the MRI signal changes, seven cases had fat within the tumor, six had bleeding, and 21 had necrosis. The detailed conventional MRI features are summarized in Table 3.

**Table 3:** Comparison of the conventional MRI characteristics of epithelioid hepatic angiomyolipoma versus hepatocellular carcinoma.

	**Epi-HAML (*n* = 30)**	**HCC (*n* = 80)**	***p***
Location			0.293
Right lobe	18 (60.0%)	49 (61.3%)	
Left lobe	10 (33.3%)	30 (37.5%)	
Caudal lobe	2 (6.7%)	1 (1.2%)	
Number			0.018
Solitary	30 (100%)	67 (83.8%)	
Multiple	0	13 (16.2%)	
Size			0.815
<3 cm	10 (33.3%)	23 (28.8%)	
≥3 cm	20 (66.7%)	57 (71.2%)	
Shape			1
Regular	22 (73.3%)	59 (73.8%)	
Irregular	8 (26.7%)	21 (26.2%)	
Border			0.027
Well-defined	28 (93.3%)	57 (71.3%)	
Ill-defined	2 (6.7%)	23 (28.7%)	
T1-weighted imaging			1
Hypointensity	29 (96.7%)	78 (97.5%)	
Isointensity	1 (3.3%)	2 (2.5%)	
T2-weighted imaging			
Homogeneous	13 (43.3%)	25 (31.3%)	0.336
Heterogeneous	17 (56.7%)	55 (68.7%)	
Isointensity	2 (6.7%)	1 (1.2%)	0.370
Hyperintensity	28 (93.3%)	79 (98.8%)	
Diffusion weighted imaging^*^			0.055
Hypointensity	2 (8.0%)	0	
Hyperintensity	23 (92.0%)	80 (100%)	
Mild	10 (40.0%)	3 (3.7%)	<0.001
Moderate	8 (32.0%)	15 (18.8%)	
Marked	5 (20.0%)	62 (77.5%)	
Fat	13 (43.3%)	7 (8.8%)	<0.001
Bleeding	2 (6.7%)	6 (7.5%)	1
Necrosis	1 (3.3%)	21 (26.3%)	0.016

* *Only 25 Epi-HAML cases underwent diffusion weighted imaging. Epi-HAML, epithelioid hepatic angiomyolipoma; HCC, hepatocellular carcinoma*.

The conventional MRI features including signal intensity on DWI sequence with high b-value and fat proportion between the Epi-HAML and HCC groups showed markedly significant differences (*P* < 0.001). Second, differences in tumor number, border, and intra-tumor necrosis between the Epi-HAML and HCC groups were statistically significant (*P* < 0.05). Additionally, differences in tumor location, size, morphology, tumor signals on T1WI and T2WI, tumor signal on DWI sequence, and intra-tumoral bleeding between the Epi-HAML and HCC groups were not statistically significant (*P* > 0.05). The detailed comparisons of conventional MRI features are summarized in Table 3.

### Contrast-Enhanced MRI Features of Epi-HAML vs. HCC

**Figure 1:**
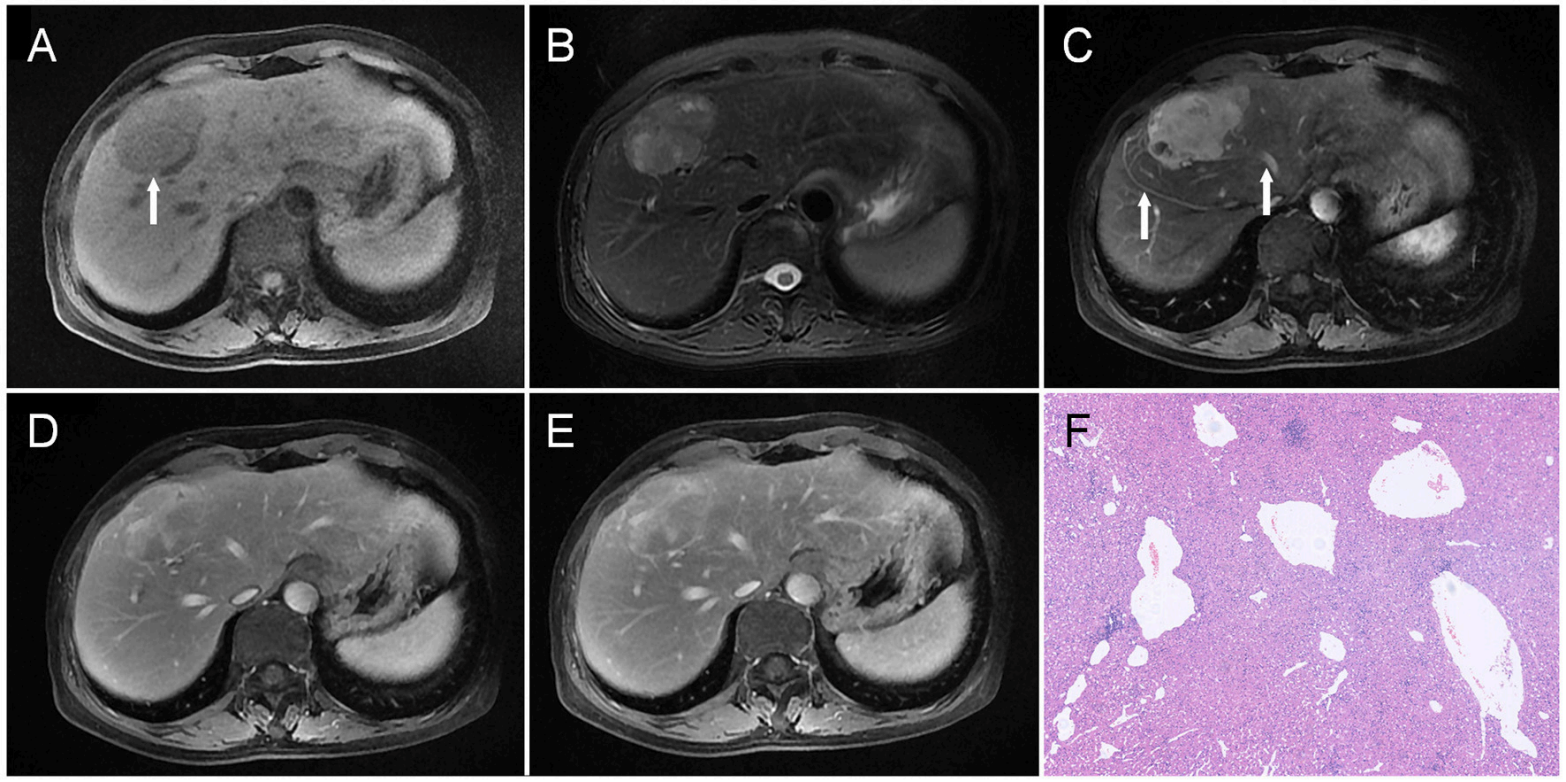
A liver mass was accidentally discovered in a 54-year-old man upon physical examination. The mass was about 5.3 cm in the diameter and was located in the junction area between the left and right lobes, which was hypointense on T1WI as indicated by an arrow in **(A)**, and heterogeneous and slightly hyperintense on T2WI **(B)**. On dynamic contrast-enhanced MRI, the tumor showed heterogeneous enhancement in the arterial phase **(C)**; besides, draining vein and hepatic vein (arrow) could also be observed. Part of the tumor maintained relative hyperintensity, which showed a prolonged enhancement pattern **(D,E)**. Besides, vascular enhancement could also be seen in the lesion (arrow, **E**). Histopathology **(F)** revealed malformed blood vessels in the tumor (HE, × 100).

**Figure 2:**
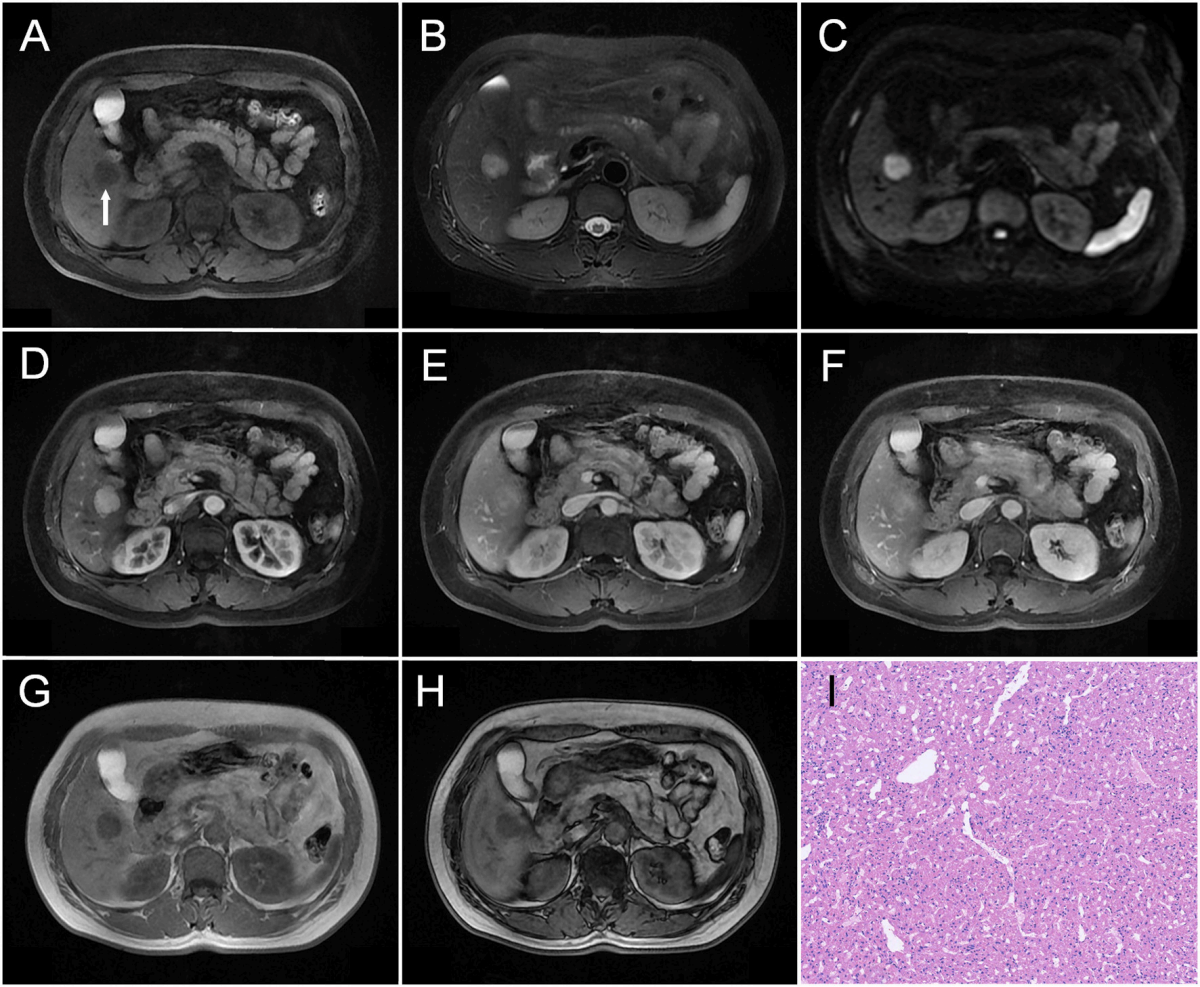
A 51-year-old woman was detected with a liver nodule during admission screening for a back lipoma. A well-defined nodule measuring about 2.1 cm in diameter was observed in the V segment of her liver as indicated by an arrow in **(A)**, which showed hypointensity on T1WI **(A)**, mildly heterogeneous hyperintensity on T2WI **(B)**, and moderately heterogeneous hyperintensity on DWI **(C)**. On dynamic contrast-enhanced MRI, the tumor was markedly enhanced on the whole during arterial phase **(D)**. During the portal **(E)** and delayed phases **(F)**, the tumor maintained the relative hyperintensity compared with the surrounding liver parenchyma. The tumor showed a prolonged enhancement pattern. No obvious signal change was observed when comparing the tumor Outphase image **(G)** with the Inphase sequence **(H)** image. Histopathological examination **(I)** revealed that the tumor was mainly constituted by epithelioid smooth muscle cells, and no obvious adipocytes were observed (HE, × 100).

**Figure 3:**
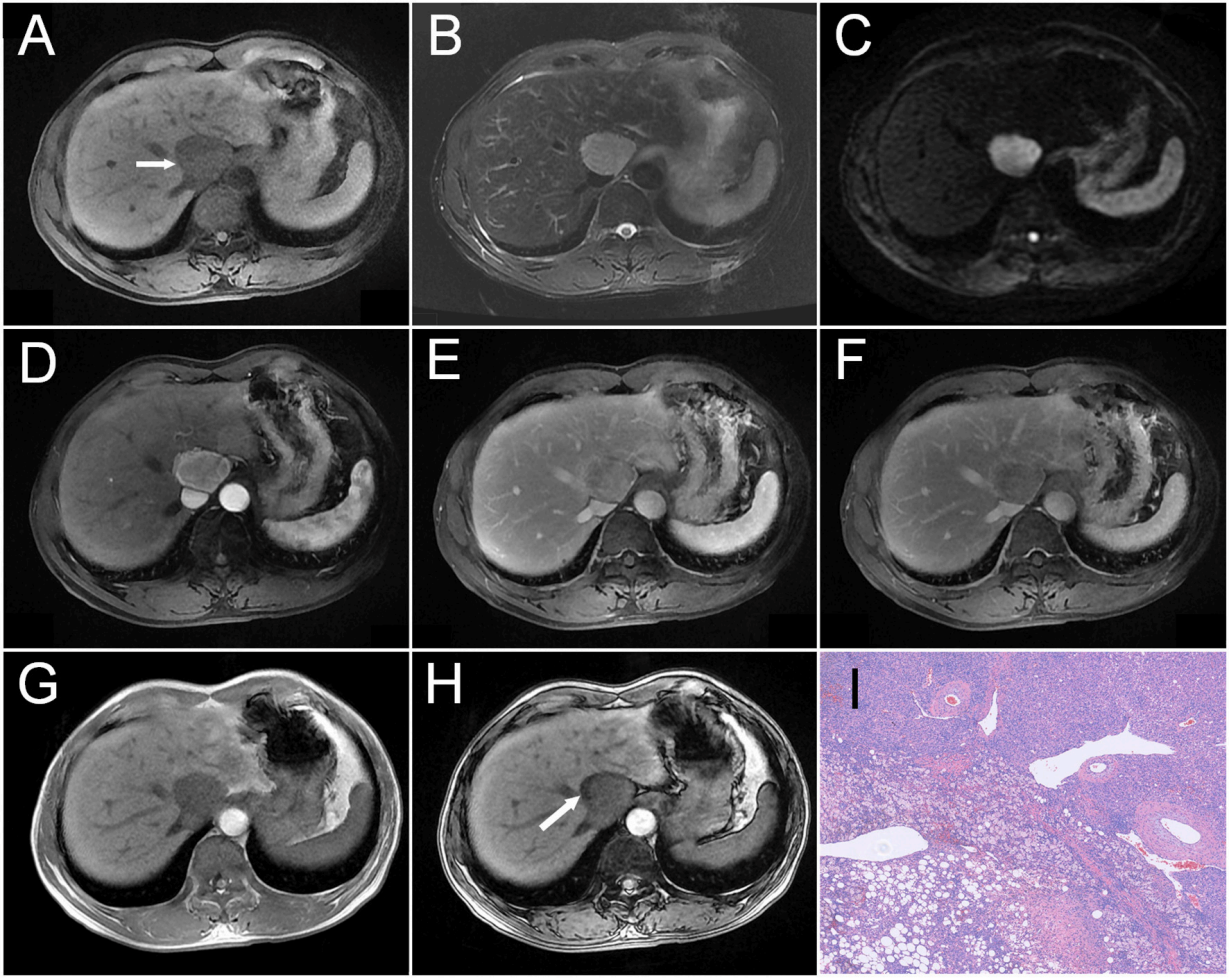
A 54-year-old man was detected with a liver mass upon physical examination. A mass measured about 4.2 cm in the diameter was located near the second hepatic portal, which showed hypointensity on T1WI as indicated by an arrow in **(A)**, slight hyperintensity on T2WI **(B)**, and heterogeneous hyperintensity on DWI **(C)**. On dynamic contrast-enhanced MRI, the tumor showed heterogeneous enhancement during the arterial phase **(D)**, as well as decreased enhancement during the portal **(E)** and delayed phases **(F)**, and showing relative hypointensity. Compared with the peripheral liver parenchyma, the tumor showed a wash-out pattern. Comparing the tumor Outphase image **(G)** with the Inphase sequence **(H)** image, locoregional signal reduction could be observed. Histopathology **(I)** revealed that the tumor was constituted by fusiform cells and vascular and adipose tissues (HE, × 100).

In the arterial phase, all 30 Epi-HAML cases showed severe hyperenhancement. Meanwhile, nine cases showed draining hepatic vein, 12 showed intra-tumor vessel, and five showed abnormal perfusion during arterial phase. In the portal phase, 14 cases showed hypoenhancement, four showed isoenhancement, while 12 showed hyperenhancement. In the delayed phase, 13 cases showed hypoenhancement, six showed isoenhancement, and 11 cases showed hyperenhancement. Additionally, 11 cases with intra-tumor vessel and six cases with capsule were confirmed during the delayed phase. As for the lesion enhancement pattern, 16 cases had prolonged enhancement (Figures 1, 2), 10 cases presented as wash out (Figure 3), and four cases showed fading. The above contrast-enhanced MRI features are summarized in Table 4.

**Table 4:** Comparison of contrast-enhanced MRI features of epithelioid hepatic angiomyolipoma vs. hepatocellular carcinoma.

	**Epi-HAML (*n* = 30)**	**HCC (*n* = 80)**	***p***
Arterial phase			0.369
Hypoenhancement	0	5 (6.3%)	
Isoenhancement	0	2 (2.5%)	
Hyperenhancement	30 (100%)	73 (91.2%)	
Draining hepatic vein	9 (30.0%)	3 (3.8%)	<0.001
Intra-tumor vessel	12 (40.0%)	32 (40.0%)	1
Abnormal perfusion	5 (16.7%)	18 (22.5%)	0.684
Portal phase			<0.001
Hypoenhancement	10 (33.3%)	60 (75.0%)	
Isoenhancement	11 (36.7%)	3 (3.8%)	
Hyperenhancement	9 (30.0%)	17 (21.3%)	
Portal venous tumor thrombosis	0	11 (13.8%)	0.033
Delayed phase			0.001
Hypoenhancement	21 (70.0%)	76 (95%)	
Isoenhancement	6 (20.0%)	2 (2.5%)	
Hyperenhancement	3 (10.0%)	2 (2.5%)	
Intra-tumor vessel	11 (36.7%)	8 (10.0%)	0.003
Pseudocapsule	6 (20.0%)	63 (78.8%)	<0.001
Pattern of enhancement			<0.001
Wash-out	7 (23.3%)	75 (93.8%)	
Prolonged enhancement	17 (56.7%)	1 (1.2%)	
Fading	6 (20.0%)	2 (2.5%)	
Poor blood supply	0	2 (2.5%)	

*Epi-HAML, epithelioid hepatic angiomyolipoma; HCC, hepatocellular carcinoma hepatic arterial phase: 14–20 s after contrast agent administration; portal phase: 40–55 s after contrast agent administration; delayed phase: 120 s after contrast agent administration*.

In the HCC control group, 73 cases manifested as hyperintense in the arterial phase, five as hypointense, and two as isointense. Moreover, three cases had draining hepatic vein, 32 had intra-tumor vessel, and 18 had abnormal perfusion at the arterial phase. At the portal phase, 60 cases showed hypointensity, three showed isointensity, 17 showed hyperintensity, and 11 had portal vein tumor thrombus. At the delayed phase, 76 cases showed hypointensity, two showed isointensity, and two showed hyperintensity. In addition, 63 had tumor pseudocapsule and eight had intra-tumor vessel at the delayed phase. As for the enhancement pattern, 75 cases manifested as wash-out, one as prolonged enhancement, two showed fading, and two had poor blood supply. The contrast-enhanced MRI features of the two groups are summarized in Table 4.

Differences in the contrast-enhanced MRI features such as draining hepatic vein in the arterial phase, tumor enhancement degree in the portal phase, intra-tumor vessel in the delayed phase, pseudocapsule, and tumor enhancement pattern between the Epi-HAML and HCC groups were markedly significant (*P* < 0.001). Second, differences in portal vein tumor thrombus and tumor enhancement degree at the venous phase between the Epi-HAML and HCC groups were statistically significant (*P* < 0.05). Moreover, differences in tumor enhancement degree, intra-tumor vessel, and abnormal perfusion in the arterial phase between the Epi-HAML and HCC groups were not statistically significant (*P* > 0.05). Comparisons of the contrast-enhanced MRI features between the two groups are summarized in Table 3.

### Pathological Findings of Epi-HAML and HCC

In the Epi-HAML group, five specimens were derived from biopsy, while 25 were from surgical resection, and all of them were definitely diagnosed as Epi-HAML. Among them, five cases were confirmed as having no definite fat component, while 19 cases had varying quantities of fat component. Additionally, there were two pathologically confirmed cases with intra-tumor bleeding and 1 with massive necrosis.

In the HCC group, all 80 cases of pathology were derived from surgically resected specimens, and all cases were diagnosed with HCC, including 21 well-differentiated, 40 moderately differentiated, and 19 poor differentiated HCCs. Among these, seven cases with fat-containing imaging signals were pathologically confirmed to have fat. Additionally, the MRI findings were also pathologically confirmed, including six cases with a small amount of intra-tumor bleeding, and 21 with intra-tumor necrosis to various degrees.

## Discussion

In our study, Epi-HAML frequently occurred in women, with the male-to-female ratio being 1:3. Typically, two-third patients showed no clinical symptoms, while the remaining one-third displayed non-specific symptoms such as abdominal pain and abdominal distention, which may not be associated with concomitant diseases (Supplementary Table 1). Similar to previous studies, Epi-HAML was more common in women, but patients with no clinical symptoms accounted for a higher proportion (17, 18, 23). In one study, 100% (6/6) cases were discovered by chance (16). In addition, in a review on Epi-HAML, female patients had taken up for about 70.0%, while those presenting symptoms occupied 35% (22). These review results were consistent with our study results, indicating that Epi-HAML was less likely to display clinical symptoms compared to reported HAML, wherein about 55% of patients with HAML displayed non-specific clinical symptoms (25). In this study, four Epi-HAML cases had concomitant hepatitis, which was likely not the related pathogenic factor, as the incidence of hepatitis was similar to that in the Chinese mainland population (26). Epi-HAML was considered to be related to the incidence of TSC, but none of our patients showed concomitant TSC, which was consistent with the existing reports in China suggesting that combined TSC was rare (6). More importantly, our study verified an important fact namely, Epi-HAML was not linked to a liver cirrhosis background, which was consistent with previous studies (22).

Our research data verified that there were significant differences in numerous clinical features between the Epi-HAML and HCC groups, which were of much value to distinguish Epi-HAML from HCC. First, Epi-HAML showed a prominent female preference, while HCC more frequently occurred in elderly men. Second, Epi-HAML had no definite correlation with HBV infection and did not occur in patients with liver cirrhosis; however, HCC was definitely correlated with HBV infection and liver cirrhosis. Third, Epi-HAML patients were generally asymptomatic given its relatively mild biological manifestations, while HCC patients were subjected to severe discomfort due to the malignant biological behavior or concomitant disease, especially in case of liver cirrhosis. Last, the abnormally elevated tumor marker AFP was only seen in HCC but not in Epi-HAML.

In our study, fat component was confirmed in about 43.3% Epi-HAML cases by means of MRI. The determination of fat was a valuable imaging diagnostic clue for Epi-HAML, which will significantly reduce the scope of differential diagnosis. Similarly, in a series of seven Epi-HAML cases, three (42.9%) had confirmed fat component on CT/MRI (17). Different from our results, in a group of six and four Epi-HAML cases, no fat component was confirmed on CT/MRI (6, 7). Additionally, in another CT study on a group of five Epi-HAML cases, only one was confirmed to have minor fatty density (19). By contrast, in another MRI study, fat was confirmed in three out of the five Epi-HAML cases (60.0%), which was apparently higher than the fat identification by CT (20).

In our opinion, the distinct difference in the fat detection proportion in Epi-HAML among studies might be related to the intratumoral heterogeneities, tumor sample size, and fat detection of the imaging approach. On the one hand, the composition proportion of Epi-HAML varied from one case to another, which would result in the individual difference in the intra-tumor fat composition, as confirmed by our pathological results. Thereby, findings related to differences in intra-tumor fat content were restricted in previous studies owing to the small Epi-HAML sample size. On the other hand, the imaging methods were also restricted in detecting the fat composition of Epi-HAML, because of the small fat content and disperse distribution of adipocytes in Epi-HAML.

On CT, the presence of fat was determined when intra-tumor density was measured to be <-20 HU. Intra-tumor fat in Epi-HAML could hardly be detected, as the insufficient number of adipocytes in a certain layer thickness would result in considerable difference in density. On MRI, multiple sequences could be used to quantify fat, including inphase and outphase, which depended on the differences in the precession frequencies of fat and water proton. It plays a more important role than CT, as it can detect small amounts of intra-tumor fat that goes undetectable in CT (27). Therefore, it was recommended that for clinically suspected Epi-HAML cases, MRI should be performed, and the appropriate sequence should be selected to improve the intra-tumor fat detection rate.

Our findings suggested that the general MRI imaging features including intra-tumor fat, necrosis ratio, and tumor signal on DWI, showed statistically significant differences between Epi-HAML and HCC groups, which contributed to distinguishing the imaging findings of the two. In our study, nearly one-half of Epi-HAML cases were confirmed with the presence of fat through the specific MRI sequence, while the presence of fat in HCC cases was notably reduced. Further, Epi-HAML was not associated with intra-tumor necrosis at all, which could be ascribed to its relatively mature vascular wall and rich blood supply. HCC, on the other hand, was likely to develop necrosis, which could be accurately evaluated through preoperative MRI images. The DWI sequence also showed a certain value in distinguishing Epi-HAML from HCC. Epi-HAML on high b-value DWI images mainly manifested as mild hyperintensity, while HCC mainly manifested as marked hyperintensity, as the intra-tumor water molecular motion was notably restricted in the latter, which had reflected the significant difference in tumor biological behaviors between the two.

Surprisingly, nine Epi-HAML cases (30%) showed early draining hepatic vein during the arterial phase and scarcely appeared in the HCC group in our study. To our best knowledge, this was the first time that the occurrence of such signs of early draining hepatic vein have been reported in a large sample size on Epi-HAML imaging study. Previously, the proportion of early draining vein was reported to be as high as 50% in a CT imaging study on fat-deficient Epi-HAML (28). Notably, only the draining hepatic vein sign in Epi-HAML was considered in our study, while the drain to hepatic vein, as well as draining portal vein and inferior vena cava, was simultaneously observed in the latter. The discovery of the early draining hepatic vein sign in Epi-HAML should be emphasized, as it was an important imaging feature to distinguish Epi-HAML from HCC (29). Moreover, the draining hepatic vein may also be well-displayed by angiography, including MRA and DSA, in patients with Epi-HAML (30, 31).

In our study, Epi-HAML generally presented as a mass rich in blood supply, and intra-tumor blood vessel could be observed in about 40% cases at different contrast-enhanced MRI phases. Different from our study, in a group of 11 HAML patients, intra-tumor blood vessel could only be observed in two (18.2%) cases, while it could not be observed in five Epi-HAML cases on MRI (20). On the contrary, in a group of Epi-HAML cases, intra-tumor blood vessel could be observed in 83.3% (5/6) cases (7). We believe that such distinct difference in the occurrence rate of intra-tumor blood vessel might be attributed to the small sample size of these two studies. Our previous study suggested that the display of intra-tumor blood vessel during non-arterial phase also contributed to the differential diagnosis with HCC, because the latter generally displayed the intra-tumor vascular enhancement during the arterial phase (29). Consequently, observing the distribution of intra-tumor blood vessel at different MRI phases might facilitate the diagnosis of Epi-HAML, given that Epi-HAML was located in the tumor center, while HCC was mostly located in the non-tumor center (18).

Pseudocapsule was confirmed in 20% Epi-HAML cases, while 63/80 HCC cases were with pseudocapsule in our MRI study. Similarly, in an imaging study of six Epi-HAML cases, only one case (16.7%) showed the presence of capsule (7). Therefore, absence of pseudocapsule might facilitate the diagnosis of Epi-HAML, as the pseudocapsule is an important diagnostic feature in HCC. Nonetheless, different from previous studies, all five Epi-HAML cases showed pseudocapsule (20). We speculated that such distinct differences in the occurrence rate of pseudocapsule between different studies might be attributed to the difference in the definition pseudocapsule among different studies. On the one hand, pseudocapsule of Epi-HAML was pathologically verified to be constituted by the squeezed surrounding liver parenchyma, while the real capsule was lacking (6). On the other hand, the non-smooth and non-integrated peripheral enhancement during portal phase and delayed phase could not be characterized as the pseudocapsule; instead, it was formed through the enhancement of peripheral blood vessels of Epi-HAML (32). Thus, in imaging analysis, the pseudocapsule appearance should be characterized with caution, especially for Epi-HAML.

In our MRI study, over a half of Epi-HAML cases (56.7%) manifested as prolonged enhancement, followed by wash-out (23.3%) and fading (20%). Thus, it was indicated that prolonged enhancement in contrast-enhanced MRI offered crucial clues for diagnosing Epi-HAML. Similar to our study, 60% (3/5) Epi-HAML cases also displayed the slow out enhancement pattern in two different contrast-enhanced imaging studies (19, 20). Different from our study, the one by Ji et al. suggested that 66.7% (4/6) Epi-HAML cases displayed hypoenhancement during the delayed phase, and only 16.7% (1/6) showed prolonged enhancement (7). Thus, about 60% HAML cases were misdiagnosed as HCC in an Epi-HAML review, owing to the wash-out pattern (6). Such diverse results in the enhancement pattern might be related to the understanding of wash-out and different imaging devices. It should be noted that when the enhancement degree of a partial area within the tumor was higher than that of the surrounding liver parenchyma during delayed phase, the enhancement pattern should not be deemed as a wash-out pattern^1^. On the other hand, the proportion of prolonged enhancement in our previous study was 40.0% on CT (29), while it was 56.7% on MRI. Hence, the reason for misdiagnosing Epi-HAML as HCC is speculated partly to the imaging modality, as intra-tumor hyperintensity could be observed more in the non-arterial phase in enhanced MRI.

It should be emphasized that the contrast-enhancement features play important irreplaceable roles in distinguishing Epi-HAML from HCC, including the two aforementioned positive features (early draining hepatic vein and intra-tumor blood vessel in the delayed phase) and one negative feature (pseudocapsule). Importantly, prolonged enhancement is common in the Epi-HAML group, which is apparently different from the wash-out pattern in the HCC group. Yet, there are still about 2/5 Epi-HAML cases that can hardly be distinguished from HCC, from the enhancement pattern alone. Particularly, for Epi-HAML with wash-out pattern, the clinical features and conventional MRI features mentioned above may be helpful to distinguish Epi-HAML from HCC. Lastly, because Epi-HAML presents as a severe enhanced mass, it should also be distinguished from other tumors with rich vasculature such as FNH, angiomas, and bile duct adenoma.

Our study has certain limitations. First, the Epi-HAML cases from two institutions had been retrospectively summarized, but the sample size was still limited because of its low incidence. Second, the DWI sequence-related ADC values of the tumor had not been quantitatively studied, as a result of the difference of imaging parameters, for example time repeat and time echo, in the two different institutions.

## Conclusions

Epi-HAML is associated with certain MRI features such as the detection of a small amount of intra-tumor fat, rich blood supply, draining hepatic vein during the arterial phase, intra-tumor vessel during the delayed phase, mild hyperintensity on DWI sequence, and the prolonged enhancement pattern. All these MRI features may be clues for Epi-HAML diagnosis and distinction from HCC.

## Author Contributions

WLiang and WLiu conception and design, development of methodology (provided animals, acquired and managed patients, provided facilities, etc.), and analysis and interpretation of data (e.g., statistical analysis, biostatistics, computational analysis). WLiang, QL, QH, WLiu, and JW writing, review, and/or revision of the manuscript and administrative, technical, or material support (i.e., reporting or organizing data, constructing databases).

### Conflict of Interest Statement

The authors declare that the research was conducted in the absence of any commercial or financial relationships that could be construed as a potential conflict of interest.

## Footnotes

^1^Available online at: https://www.acr.org/Clinical-Resources/Reporting-and-Data-Systems/LI-RADS
