# Supplementary material for: Comparison of MRI Features of Epithelioid Hepatic Angiomyolipoma and Hepatocellular Carcinoma: Imaging Data From Two Centers
Source: Front Oncol. 2018 Dec 12;8:600. doi: 10.3389/fonc.2018.00600 (PMC6299843; doi:10.3389/fonc.2018.00600)
Supplement: Supplementary file 1 [file Data_Sheet_1.docx]

Supplementary material

Ⅰ. The schematic of liver lobe


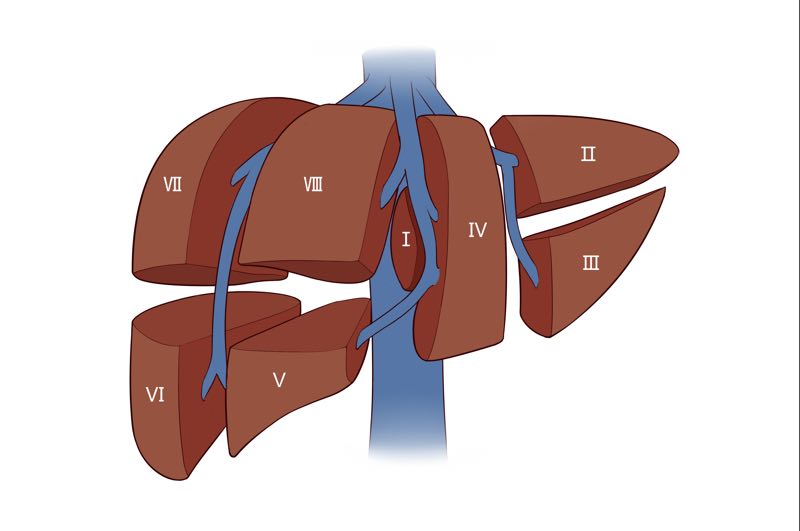


Supplementary Figure 1. The schematic of liver lobe.
I: caudal lobe, II-IV: left lobe, V-Ⅷ: right lobe

Ⅱ. Supplementary Table 1. The comparison of incidence of concomitant disease between symptomatic Epi-HAML and asymptomatic Epi-HAML

|  | Symptomatic Epi-HAML (9) | Asymptomatic Epi-HAML (21) | *p* |
| --- | --- | --- | --- |
| Concomitant disease |  |  |  |
| Hepatitis B | 1(11.1%) | 3(14.3%) | 1.000 |
| Fatty liver disease | 2(22.2%) | 2(9.5%) | 0.563 |
| Cholelithiasis | 0(0) | 1(4.8%) | 1.000 |
| Hypertension | 1(11.1%) | 2(9.5%) | 1.000 |
| SPSS (Version 23.0; SPSS Inc., Chicago, IL, USA) was used, Fisher's exact test was used to compare the two groups. | | | |

The statistical results indicate that there is no significant difference in the concomitant disease between symptomatic and asymptomatic Epi-HAML, and symptoms may not be markedly correlated with concomitant disease.
